# Antenatal Pulmonary Hypoplasia Expands the Variety of Phenotypes Associated With *MED12*‐Related Disorders

**DOI:** 10.1002/pd.6881

**Published:** 2025-08-30

**Authors:** Vijaya Ramachandran, Natalie J. Chandler, Alexander Gibbs, Elspeth Whitby, Ruth Braham, Joseph Christopher, Sarju G. Mehta

**Affiliations:** ^1^ NHS North Thames Genomic Laboratory Hub Great Ormond Street Hospital for Children NHS Foundation Trust London UK; ^2^ Genetics and Genomic Medicine UCL Great Ormond Street Institute of Child Health London UK; ^3^ Sheffield Teaching Hospitals NHS Foundation Trust Sheffield UK; ^4^ Addenbrooke's Hospital Cambridge UK

**Keywords:** female fetus, hardikar syndrome, *MED12*, prenatal diagnosis, pulmonary hypoplasia, X‐linked

## Abstract

- What's already known about this topic?
  ◦ Hardikar Syndrome is one of the syndromes caused by *MED12* germline pathogenic variants and is associated with varying levels of neurocognitive disability. Prenatal features of Hardikar Syndrome previously identified include cleft palate and diaphragmatic herniation.
- What does this study add?
  ◦ To the best of our knowledge, this is the first reported case of a fetus with Hardikar syndrome demonstrating pulmonary hypoplasia secondary to diaphragmatic hernia.

## 1 Report

Rapid prenatal trio exome testing was requested for a female fetus following the detection of fetal anomalies during fetal ultrasound scanning.

### 1.1 Fetal Phenotype

First trimester routine ultrasound scanning showed increased nuchal translucency of 7.2 mm and cystic hygroma. An early second trimester anatomy scan demonstrated subcutaneous lower body edema, bilateral cleft lip and palate, head circumference on the third centile, and a posteriorly positioned stomach with the cardiac axis shifted right, indicative of diaphragmatic hernia (see Table 1).

**TABLE 1:** Clinical data.

Case	Parental details			Gestation at diagnosis	Phenotypes (HPO terms)	Obstetric history	Family history	Outcome
1	Maternal	Age	28 years	16 + 4	Increased nuchal translucency (HP:0,010,880), cystic hygroma (HP:0,000,476), bilateral cleft lip and palate (HP:0,002,744), central diaphragmatic hernia (HP:0,025,195)	Four previous live births, all well	Nil	Termination of pregnancy at 26 + 4 weeks
		Ethnicity	White British					
	Paternal	Age	27 years					
		Ethnicity	Not given					

### 1.2 Diagnostic Method

Fetal DNA was extracted from cultured amniocytes. Initial QF‐PCR for common trisomies and microarray were unremarkable. Rapid prenatal trio exome sequencing on fetal DNA extracted from cultured amniocytes and parental blood samples with analysis using a fetal anomalies panel was carried out as previously described [1].

### 1.3 Diagnostic Results and Interpretation

The female fetus was found to be heterozygous for an apparently *de novo MED12* c.99+2T > G likely pathogenic variant (see Table 2). This sequence change affects a donor splice site in intron 1 of the *MED12* gene and is expected to disrupt RNA splicing of exon 1, resulting in the loss of the initiation codon. There is only one annotated transcript for this gene; therefore, this variant is predicted to cause loss of function, which is a known mechanism of pathogenicity for *MED12* variants [2, 3]. This variant is novel and absent from the gnomAD population database. Sanger sequencing confirmed the variant. No functional or splice studies were carried out.

**TABLE 2:** Genetic findings.

Procedure (gest age)	Direct/culture?	Performed test	Secondary confirmatory test	Gene (name; REFSEQ)	Known disease (OMIM)	Variant	ACMG classify‐cation	Criteria applied	Inheritance & zygosity	Interpret‐ation
16 + 4	Amniocytes ‐ culture	Prenatal trio exome	Sanger sequencing	*MED12* (NM_005120.2)	Hardikar syndrome (301,068; XLD) Lujan‐Fryns syndrome (309,520; XLR) Ohdo syndrome, X‐linked (300,895; XLR) Opitz‐Kaveggia syndrome (305,450; XLR)	c.99+2T > G	Likely pathogenic	PVS1_strong, PM2_mod, PS2_moderate	Heterozygous; *de novo* (female fetus)	Contributes to fetal presentation

Germline pathogenic variants within the *MED12* gene lead to a range of phenotypes with overlapping features including Hardikar syndrome (MIM 301068; XLD), Lujan‐Fryns syndrome (MIM 309520; XLR), X‐linked Ohdo syndrome (MIM 300895; XLR), Opitz‐Kaveggia syndrome/FG syndrome‐1 (MIM 305450; XLR) and nonspecific intellectual disability [3]. Specific missense variants are associated with FG syndrome‐1, Lujan‐Fryns syndrome, X‐linked Ohdo syndrome, nonspecific intellectual disability cause disease in hemizygous males. Hemizygous loss of function variants are assumed to be lethal, which is supported by studies in mice [4]. Loss of function variants in females have been reported both with Hardikar syndrome [2, 5] and nonspecific intellectual disability [3] with a spectrum of neurodevelopmental disability ranging from normal [2] to severe [3]. A likely cause of phenotypic variability is X‐inactivation, which can differ across tissues [4, 5]. The prenatal features along with the type of variant in this fetus were felt to be indicative of Hardikar syndrome. There remained uncertainty as to the likely neurocognitive outcome for this fetus, which presents counseling challenges. The variant was reported at 23 + 3 weeks gestation as likely pathogenic.

### 1.4 Pregnancy Outcome

**FIGURE 1:**
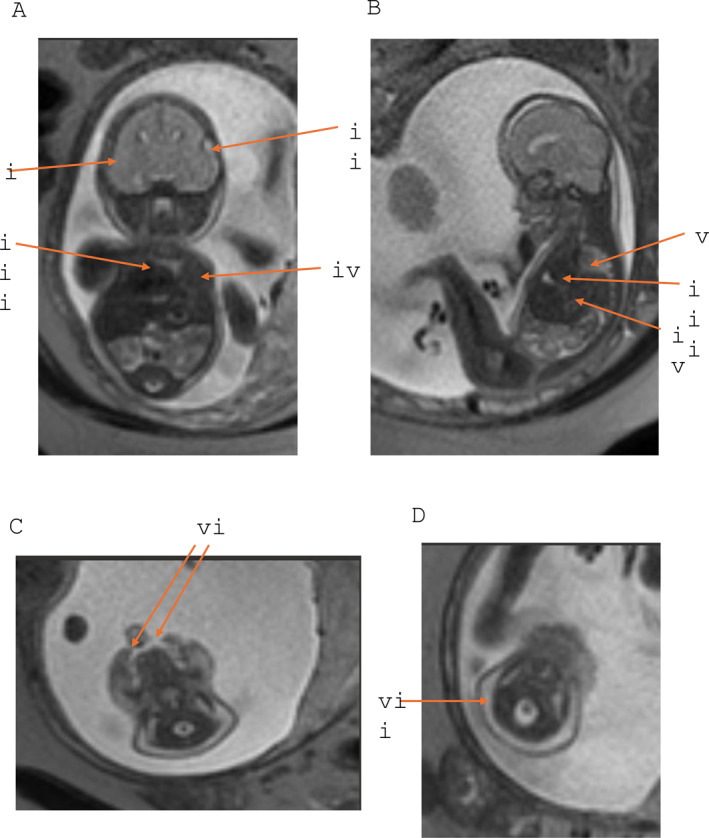
A–D: Fetal MRI images. Figure 1A is a coronal plane view of the head and brain showing turricephalic head (i) and significantly reduced extra axial CSF space (ii). Figure 1B is a sagittal plane view. The two views demonstrate a right shifted heart (iii) and liver herniation into the chest cavity (iv). Hypoplastic lung (v) due to the herniating liver is shown in Figure 1B. Figure 1C is an axial view of the fetal head demonstrating a cleft palate (vi). Figure 1D is an axial view of the fetal neck and inferior head displaying the cystic hygroma (vii).

Fetal echocardiogram performed at 24 + 2 weeks gestation showed a structurally normal albeit right‐shifted heart. Fetal MRI at 24 + 4 weeks (see Figure 1A–D) showed microcephaly with dolichocephalic and turricephalic changes and significantly reduced extra axial CSF space, bilateral cleft lip and palate, cystic hygroma, highly unusual thoracic appearance with herniation of liver and stomach into thoracic cavity, lung volume 11% of expected and low signal intensity suggesting pulmonary hypoplasia (absence of lower trachea and bifurcation). The severity of the lung hypoplasia in this fetus was almost certainly incompatible with *ex utero* life and the parents made the difficult decision to proceed with feticide and termination of pregnancy at 26 + 4 weeks gestation. As this variant was *de novo,* the parents were counseled that the recurrence risk was low.

### 1.5 Discussion

Some of the features observed in this case overlap with other *MED12*‐related disorders rather than Hardikar syndrome, including skull abnormalities (dolichocephaly and turricephaly) and microcephaly. It is unclear whether these disorders are simply spectrum or distinct allelic disorders. To the best of our knowledge, pulmonary hypoplasia has not been previously reported in postnatal cases with *MED12* variants. However, diaphragmatic hernia is a previously reported finding in Hardikar syndrome [4, 6] and pulmonary hypoplasia secondary to diaphragmatic aplasia has been reported in a female fetus with a *MED12* loss of function variant [7]. Therefore, this finding provides further evidence for inclusion of pulmonary hypoplasia as part of the *MED12*‐phenotypic spectrum.

This case demonstrates the challenges of determining the likely postnatal phenotype from a prenatal finding of a germline pathogenic variant in the *MED12* gene due to the large range of potential phenotypic outcomes. It also provides further evidence to expand the phenotypic spectrum of *MED12*‐related disorders to include pulmonary hypoplasia.

## Ethics Statement

The authors have nothing to report.

## Consent

Written parental consent was obtained to share the details of this case.

## Conflicts of Interest

The authors declare no conflicts of interest.

## Data Availability

The data that supports the findings of this report are available from the authors upon a reasonable request.
